# Feasibility of EuroQoL-5 Dimensions (EQ-5D) and EuroQoL Visual Analogue Scale (EQ-VAS) Assessment in Routine Postoperative Follow-Up After Surgical and Transcatheter Heart Valve Interventions: A Pragmatic Cross-Sectional Pilot Study

**DOI:** 10.7759/cureus.105117

**Published:** 2026-03-12

**Authors:** Andra D Marinescu, Andreea Costache, Stefan A Oprea, Victor S Costache

**Affiliations:** 1 Pulmonology, Faculty of Medicine, Titu Maiorescu University, Bucharest, ROU; 2 Cardiovascular Surgery, Sanador Clinical Hospital, Bucharest, ROU; 3 Cardiovascular Surgery, Faculty of Medicine, Titu Maiorescu University, Bucharest, ROU

**Keywords:** eq-5d-5l, feasibility study, heart valve interventions, patient-reported outcomes, quality of life, transcatheter valve therapy

## Abstract

Background

Health-related quality of life (HRQoL) has become an essential patient-centered outcome in modern cardiovascular care, particularly following heart valve interventions. Although advances in surgical and transcatheter therapies have markedly improved survival, real-world implementation of standardized patient-reported outcome measures (PROMs) remains limited due to feasibility concerns. This study aimed to evaluate the feasibility of EQ-5D (EuroQoL-5 Dimensions) and EQ-VAS (EuroQoL Visual Analogue Scale) implementation in routine postoperative follow-up and to explore associations between HRQoL and selected clinical variables.

Methods

This single-center cross-sectional pilot feasibility study included consecutive adult patients who had undergone surgical or transcatheter heart valve interventions and attended routine postoperative follow-up approximately two to four months after intervention. HRQoL was assessed using the EQ-5D questionnaire and EQ-VAS. A Global Rating of Change question evaluated perceived health status relative to the pre-intervention period. Clinical and procedural data were extracted retrospectively from medical records. Feasibility outcomes included completion rate, independence of questionnaire completion, and absence of missing data. Associations between EQ-VAS scores and clinical variables were explored using nonparametric analyses.

Results

Among 40 eligible patients, 34 (85.0%) completed the HRQoL assessment without missing responses or needing assistance, demonstrating excellent feasibility. The mean EQ-VAS score was 79.4 ± 13.2. Overall, 12 (35.3%) of patients reported full health status across all EQ-5D domains. Functional limitations were uncommon, with mobility impairment reported by 3 (8.8%) patients and self-care and usual activity limitations each by 5 (14.7%), while pain/discomfort (11, or 32.4%) and anxiety/depression (9, or 26.5%) were the most frequent concerns. Most patients, 28 (82.4%), perceived their health as improved compared to the pre-intervention period. Clinically significant postoperative complications occurred in 2 (5.9%) patients and were associated with significantly lower EQ-VAS scores (p = 0.046).

Conclusions

Implementation of EQ-5D and EQ-VAS in routine postoperative follow-up after heart valve interventions is feasible, well accepted by patients, and provides meaningful insights into perceived recovery. These findings support the integration of pragmatic HRQoL assessment into real-world cardiovascular practice and provide a foundation for future longitudinal studies evaluating the role of patient-reported outcomes in guiding patient-centered care.

## Introduction

Health-related quality of life (HRQoL) has emerged as a critical outcome measure in modern cardiovascular medicine, particularly in patients undergoing heart valve interventions [1,2]. Advances in surgical techniques, minimally invasive procedures, and transcatheter therapies have substantially improved survival rates, shifting clinical focus toward patient-centered outcomes such as functional recovery, symptom burden, and overall well-being [3].

Traditional clinical endpoints, including mortality, complication rates, and echocardiographic parameters, provide essential information regarding procedural success but do not fully capture the patient’s subjective experience following intervention [4]. Consequently, patient-reported outcome measures (PROMs) have gained increasing importance in both research and routine clinical practice [5].

Among PROMs, the EQ-5D questionnaire is one of the most widely validated and internationally recommended instruments for assessing HRQoL [6]. It offers standardized evaluation across five domains: mobility, self-care, usual activities, pain/discomfort, and anxiety/depression. The EQ-VAS component provides a simple global health rating on a 0-100 scale [6,7]. Although widely used, generic PROM instruments such as EQ-5D may have limitations when applied to specific cardiovascular populations. Their broad domain structure may be susceptible to ceiling effects and may not capture subtle disease-specific symptoms such as exertional dyspnea, reduced exercise tolerance, or valvular fatigue. Furthermore, global self-rated health measures such as EQ-VAS are inherently subjective and may be influenced by psychological adaptation, social context, or non-cardiac comorbidities [6,7].

Despite its established validity, implementation of EQ-5D in routine postoperative cardiovascular settings remains inconsistent, often due to concerns regarding feasibility, workflow integration, and patient compliance [8]. In pragmatic clinical environments, there is a growing need for rapid, easily administered PROM tools that can be seamlessly incorporated into follow-up visits [9].

Therefore, the primary objective of this pilot study was to assess the feasibility and patient acceptability of implementing EQ-5D and EQ-VAS in routine postoperative follow-up after heart valve interventions. The secondary exploratory objective was to describe postoperative HRQoL profiles and examine whether EQ-VAS scores showed preliminary associations with selected clinical indicators. The study was not designed to establish prognostic validity or compare treatment strategies but rather to evaluate whether systematic collection of PROMs could be practically integrated into routine clinical workflows.

## Materials and methods

This study was designed as a single-center, cross-sectional pilot feasibility investigation conducted at a tertiary cardiovascular center. The present analysis represents a predefined exploratory component of a broader ongoing doctoral research project focused on long-term quality of life outcomes in patients undergoing heart valve interventions. Within this larger framework, the current study aimed specifically to evaluate the practical feasibility, patient acceptability, and real-world applicability of implementing standardized patient-reported outcome measures in routine postoperative follow-up.

Eligible participants were consecutive adult patients who had previously undergone surgical or transcatheter heart valve interventions at the study institution and who presented for routine postoperative clinical evaluation. Health-related quality of life (HRQoL) assessment was performed at a single postoperative time point, typically between two and four months after the intervention, corresponding to a period of early functional recovery when patients had completed the immediate postoperative phase but remained in active clinical follow-up.

Inclusion criteria consisted of age ≥18 years, prior surgical or transcatheter heart valve intervention, clinical stability at follow-up, and the ability to provide informed consent and independently complete the questionnaire. Patients were excluded if they had severe cognitive impairment, significant communication limitations, or acute clinical instability that could interfere with reliable questionnaire completion.

Health-related quality of life was assessed using the EQ-5D (the Euro Quality of Life 5 Dimensions) instrument, a widely validated and internationally recommended patient-reported outcome measure [10]. The questionnaire evaluates five health domains: mobility, self-care, usual activities, pain/discomfort, and anxiety/depression, each rated on a five-level severity scale. In addition, patients completed the EQ-VAS (Euro Quality of Life Visual Analogue Scale), a visual analog scale ranging from 0 (worst imaginable health state) to 100 (best imaginable health state), providing a global self-assessment of perceived health status [11]. The study protocol was approved by the Ethics Committee of Titu Maiorescu University (Approval No. 21/06/11/2025) and conducted in accordance with the principles of the Declaration of Helsinki. Informed consent was obtained from all participants prior to enrollment.

Because baseline pre-intervention HRQoL data were not systematically available within routine clinical records, a supplementary single-item Global Rating of Change question was included to capture patients’ subjective perception of their current health relative to their preoperative condition. This retrospective self-assessment inherently introduces potential recall bias and response shift and, therefore, should be interpreted only as a contextual patient perspective rather than as a substitute for prospective baseline measurement. However, it provides meaningful contextual information in pragmatic real-world settings where prospective baseline PROM (patient-reported outcome measures) collection is not routinely implemented.

Clinical and procedural data were extracted retrospectively from institutional electronic medical records. These included demographic characteristics, New York Heart Association (NYHA) functional class, EuroSCORE II risk assessment [12], length of hospital stays, intensive care unit admission, and clinically significant postoperative complications. Postoperative complications were defined as events requiring therapeutic intervention, including permanent pacemaker implantation or prolonged mechanical ventilation.

Feasibility outcomes were evaluated based on the proportion of eligible patients who successfully completed the EQ-5D and EQ-VAS, the absence of missing responses, and the ability of patients to complete the questionnaires independently without assistance. These measures were chosen to reflect real-world implementation practicality rather than purely psychometric performance.

Statistical analysis was primarily descriptive, consistent with the exploratory pilot nature of the study. Continuous variables were expressed as mean ± standard deviation, and categorical variables as frequencies and percentages. Associations between EQ-VAS scores and selected clinical variables were explored using nonparametric correlation and group comparison analyses. Given the limited sample size, all association analyses should be interpreted as exploratory and hypothesis-generating rather than confirmatory. No multivariable modeling was performed. A two-sided p-value <0.05 was considered statistically significant. All analyses were conducted using R statistical software [13].

## Results

Study population and clinical characteristics

A total of 40 consecutive patients who had undergone surgical or transcatheter heart valve interventions were screened for inclusion. Of these, 34 patients (85.0%) successfully completed the health-related quality of life assessment and were included in the final analysis.

The mean age of the study cohort was 62.3 ± 14.5 years, and the majority of participants, 24 (70.6%), were male. Most patients, 29 (85.3%), were classified as NYHA functional class II at the time of assessment, reflecting overall clinical stability during follow-up. The mean EuroSCORE II was 2.4 ± 3.1, indicating generally low operative risk within the cohort.

Regarding procedural strategy, transcatheter interventions represented the largest group, accounting for 18 patients (52.9%), including 17 transcatheter aortic valve implantations and one MitraClip procedure. Minimally invasive surgical approaches were performed in 14 patients (41.2%), while conventional sternotomy was required in two patients (5.9%), including one planned sternotomy and one intraoperative conversion from a minimally invasive approach due to technical difficulties.

Clinically significant postoperative complications were observed in two patients (5.9%), consisting of one permanent pacemaker implantation and one case of prolonged mechanical ventilation exceeding 24 hours. Clinical characteristics are summarized in Table 1.

**Table 1:** Baseline Clinical and Procedural Characteristics of the Study Population Mean ± SD; n (%), BMI: Body mass index, COPD: Chronic obstructive pulmonary disease, NYHA: New York Heart Association, EQ-VAS: EuroQol Visual Analogue Scale

	Overall (n = 34)
Variable	N = 34
Age	62.3 ± 14.5
Sex	
F	10 (29.4%)
M	24 (70.6%)
BMI	26.1 ± 3.9
Arterial hypertension	31 (91.2%)
Hypercholesterolemia	30 (88.2%)
Diabetes (type II)	6 (17.6%)
COPD	0 (0.0%)
NYHA class	
I	1 (2.9%)
II	29 (85.3%)
III	4 (11.8%)
IV	0 (0.0%)
EUROSCORE II	2.4 ± 3.1
Any complication (post-op)	2 (5.9%)
EQ-VAS	
50	5 (14.7%)
60	0 (0.0%)
70	0 (0.0%)
80	16 (47.1%)
90	13 (38.2%)

Feasibility of HRQoL assessment

Among the 40 eligible patients approached during routine postoperative follow-up, 34 completed the EQ-5D and EQ-VAS questionnaires, corresponding to a completion rate of 85.0%. Among completed questionnaires, there were no missing responses, and all respondents completed the assessment independently without assistance. 

These findings suggest that administration of these questionnaires was operationally feasible within this selected outpatient pilot setting. However, because the study population consisted of clinically stable patients able to attend follow-up visits and complete questionnaires independently, these feasibility estimates may not necessarily reflect implementation in broader or higher-acuity patient populations. These findings indicate excellent feasibility and acceptability of implementing standardized patient-reported outcome measures within routine postoperative clinical practice.

Health-related quality of life outcomes

The mean EQ-VAS score was 79.4 ± 13.2, with values ranging from 50 to 90, reflecting generally favorable perceived health status in the early postoperative period.

A total of 12 patients (35.3%) reported full health status across all EQ-5D domains.

Functional impairments were relatively uncommon. Mobility limitations were reported by 3 patients (8.8%), while difficulties in self-care and usual activities were each reported by 5 patients (14.7%). In contrast, symptom-related domains demonstrated a higher burden, with pain/discomfort reported by 11 patients (32.4%) and anxiety/depression by 9 patients (26.5%). 

When asked to compare their current health status with their condition prior to intervention, most patients reported substantial improvement. Overall, 28 patients (82.4%) perceived their health as improved, 5 patients (14.7%) reported no change, and only one patient (2.9%) reported worsening.

HRQoL outcomes are summarized in Table 2.

**Table 2:** Health-Related Quality of Life Outcomes EQ-VAS: EuroQol Visual Analogue Scale

Outcome	Value
EQ-VAS (mean ± SD)	79.4 ± 13.2
EQ-VAS range	50–90
Patients reporting no issues	12 (35.5%)
Mobility problems	3 (8.8%)
Self-care problems	5 (14.7%)
Usual activities problems	5 (14.7%)
Pain/Discomfort	11 (32.4%)
Anxiety/Depression	9 (26.5%)
Self-perceived health improvement vs pre-intervention	
Better	28 (82.4%)
Same	5 (14.7%)
Worse	1 (2.9%)

Associations between HRQoL and clinical variables

EQ-VAS scores showed no significant correlation with NYHA functional class (Spearman rho = 0.03, p = 0.862) or EuroSCORE II (rho = 0.08, p = 0.658).

Patients who experienced clinically significant postoperative complications demonstrated lower EQ-VAS scores compared to those without complications (p = 0.046). However, because only two patients experienced such complications, this association should be interpreted with substantial caution and viewed as exploratory rather than confirmatory. This can be seen in Table 3.

**Table 3:** Correlation and Association Analyses for EQ-VAS Scores rho (ρ): Spearman correlation coefficient, *: indicates statistical significance (p < 0.05)

Analysis	Estimate	p-value
EQ-VAS vs. NYHA (Spearman)	rho = 0.03	0.862
EQ-VAS vs. EuroSCORE II (Spearman)	rho = 0.08	0.658
EQ-VAS vs. complications (Wilcoxon)	—	0.046*

## Discussion

This pilot feasibility study suggests that systematic administration of EQ-5D and EQ-VAS questionnaires during routine postoperative follow-up after heart valve interventions is operationally achievable in a selected cohort of clinically stable patients attending outpatient evaluation. The high completion rate, absence of missing responses among completed questionnaires, and independent completion by all respondents indicate that these instruments can be incorporated into routine follow-up visits in this specific setting [14,15]. However, the feasibility observed in this limited context should not be interpreted as evidence of universal implementation readiness across broader or more heterogeneous cardiovascular populations.

In contemporary cardiovascular practice, advances in surgical techniques and transcatheter therapies have significantly improved survival outcomes in patients with valvular heart disease [16]. Consequently, clinical priorities have increasingly shifted toward patient-centered outcomes, including functional recovery, symptom burden, and overall quality of life. Traditional clinical metrics, although essential, often fail to fully capture the patient’s subjective recovery experience following intervention [17,18]. Our findings support the role of EQ-VAS as a rapid and sensitive global indicator of perceived health status in the early postoperative period.

The overall HRQoL profile observed in this cohort was favorable, with relatively high EQ-VAS scores and a substantial proportion of patients reporting full health status across EQ-5D domains. Functional limitations were infrequent, whereas symptom-related domains, particularly pain/discomfort and anxiety/depression, were more commonly affected. This pattern is consistent with previous studies of postoperative valve populations, which have shown that physical functional recovery typically precedes complete resolution of subjective symptom burden and psychological distress [19,20].

Importantly, most patients reported a subjective improvement in health status compared to the pre-intervention period. This finding provides strong face validity for the observed EQ-VAS results and supports the clinical effectiveness of contemporary valve interventions from a patient-centered perspective. Similar improvements in patient-reported health status following valve interventions have been reported in both surgical and transcatheter cohorts [21]. The favorable HRQoL profile observed in this cohort should also be interpreted cautiously. EQ-5D is a generic health status instrument and may be susceptible to ceiling effects, particularly in relatively well-recovered populations. Consequently, the absence of problems across EQ-5D domains does not necessarily imply complete physiological restoration, as more subtle cardiovascular limitations such as exertional dyspnea or reduced aerobic capacity may persist despite preserved basic independence. Disease-specific instruments, such as the Kansas City Cardiomyopathy Questionnaire [22], may offer greater sensitivity to these limitations and could complement generic PROM measures in future studies.

A key finding of this study is the significant association between lower EQ-VAS scores and the occurrence of clinically significant postoperative complications. This observation may suggest that adverse clinical events influence a patient’s perceived recovery. However, this finding must be interpreted cautiously because only two complication events occurred in the study cohort. As such, the statistical association observed should be viewed as hypothesis-generating rather than as evidence of a reproducible clinical relationship. Still, previous research has similarly shown that PROMs can capture the functional and psychosocial impact of complications more effectively than traditional clinical endpoints alone [23].

Notably, no significant associations were observed between EQ-VAS scores and NYHA functional class or EuroSCORE II. This lack of correlation likely reflects the different conceptual domains captured by these measures. While NYHA class and surgical risk scores primarily assess physiological disease severity and operative risk, EQ-VAS reflects subjective health perception influenced by broader biopsychosocial factors. This divergence highlights the complementary, rather than overlapping, role of PROMs in postoperative assessment [24].

An important strength of this study lies in its pragmatic design. Unlike controlled clinical trials with standardized follow-up intervals, HRQoL assessment was performed within routine clinical practice at variable postoperative time points. This approach enhances external validity and mirrors real-world clinical conditions in which PROM implementation would occur. Furthermore, the study population reflects contemporary valve practice, characterized by a predominance of minimally invasive and transcatheter interventions.

Limitations

Several limitations should be acknowledged. First, this was a small single-center pilot study, and the limited sample size restricts statistical precision and generalizability. The findings should therefore be interpreted as preliminary and context-specific. 

Second, the cross-sectional design provides only a single postoperative snapshot and does not allow assessment of longitudinal recovery trajectories, minimum clinically important change, or long-term prognostic value. 

Third, baseline pre-intervention HRQoL data were not available. The Global Rating of Change question was included only to provide a contextual patient perspective, but it is vulnerable to recall bias and response shift, and cannot substitute for prospective baseline measurement.

Fourth, clinical and procedural data were extracted retrospectively from medical records, which may limit the completeness and standardization of baseline information. 

Fifth, the study population consisted of patients who were clinically stable and able to attend outpatient follow-up and complete questionnaires independently. This introduces potential survivor and selection bias and may lead to more favorable HRQoL profiles and feasibility estimates than would be expected in a broader postoperative valve population. 

Sixth, feasibility was assessed primarily through questionnaire completion metrics. The study did not formally evaluate staff workload, cost, sustainability, electronic health record integration, or the influence of PROM data on clinical decision-making. 

Finally, EQ-5D and EQ-VAS are generic health status instruments and may not fully capture disease-specific cardiovascular symptoms. Ceiling effects and limited sensitivity to subtle functional limitations should therefore be considered when interpreting the findings.

Implications and future directions

Despite these limitations, this study provides important preliminary evidence supporting the feasibility of integrating standardized HRQoL assessment into routine cardiovascular practices. The findings suggest that PROMs can be efficiently implemented without disrupting clinical workflows and can provide clinically meaningful insights into patient recovery.

Future research should focus on longitudinal PROM monitoring, integration of HRQoL data into clinical decision-making, and exploration of predictive models linking PROM outcomes with long-term clinical endpoints.

## Conclusions

In this single-center pragmatic pilot study, administration of EQ-5D and EQ-VAS during routine postoperative follow-up after heart valve interventions was feasible in a selected group of clinically stable patients. Participants were able to complete the questionnaires independently with a high completion rate and no missing responses among completed forms. The study provides preliminary descriptive information regarding patient-reported postoperative health status but does not establish prognostic utility or broad clinical applicability. Because of the small sample size, cross-sectional design, absence of baseline HRQoL measurements, and selection of stable follow-up attendees, the findings should be interpreted cautiously. 

Larger prospective longitudinal studies incorporating baseline assessments, standardized follow-up intervals, and disease-specific PROM instruments are needed to more clearly define the role of patient-reported outcomes in postoperative valvular care.
